# Supplementary figures and images for: Co-Targeting MAP Kinase and Pi3K-Akt-mTOR Pathways in Meningioma: Preclinical Study of Alpelisib and Trametinib
Source: Cancers (Basel). 2022 Sep 13;14(18):4448. doi: 10.3390/cancers14184448 (PMC9496760; doi:10.3390/cancers14184448)

Figure 1

BEN-MEN-1

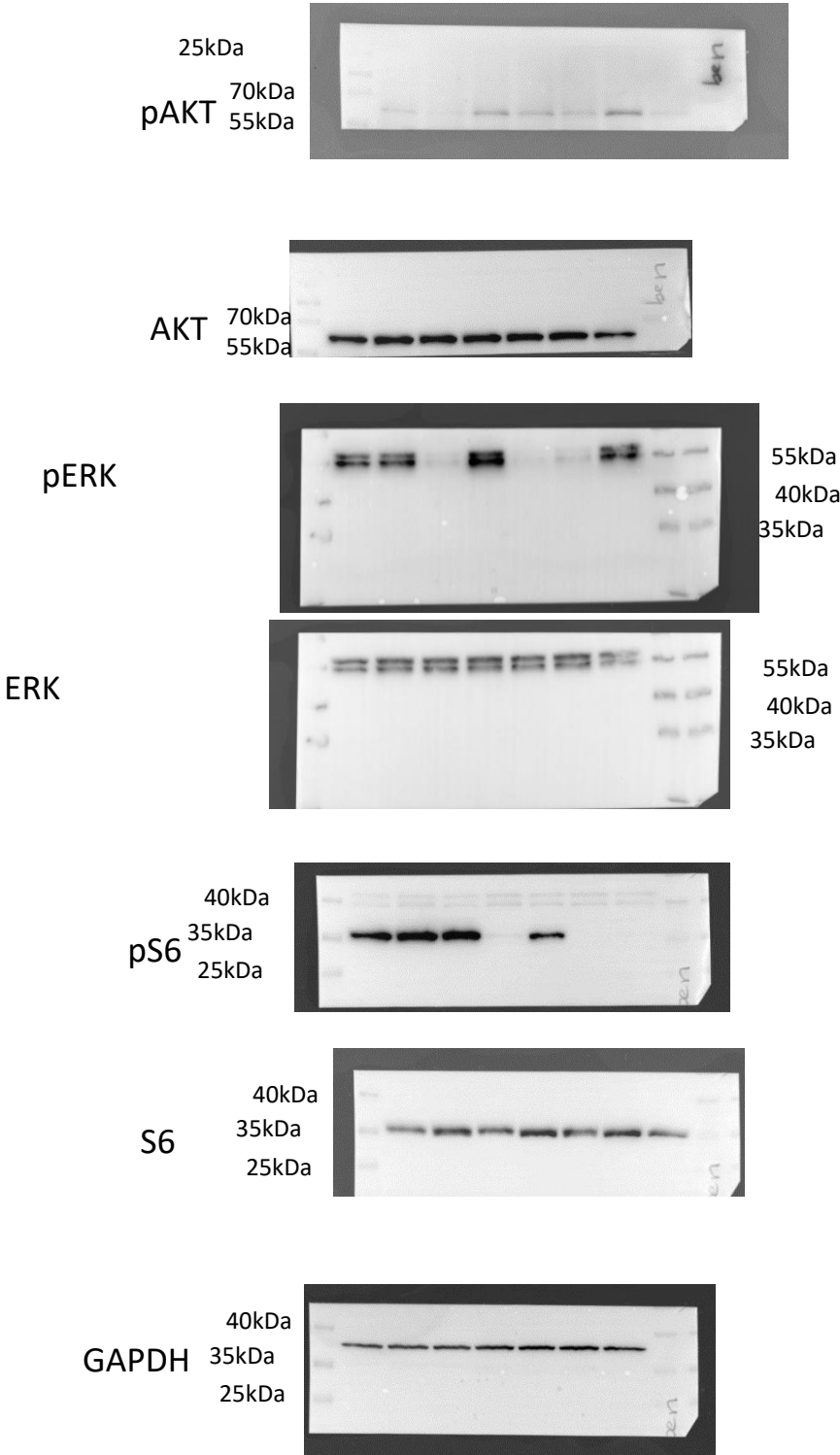

# CH-157MN

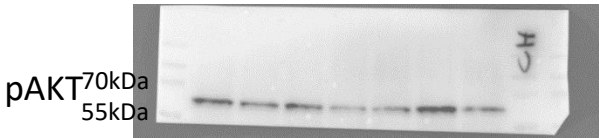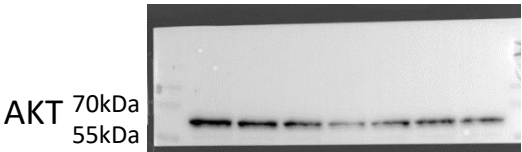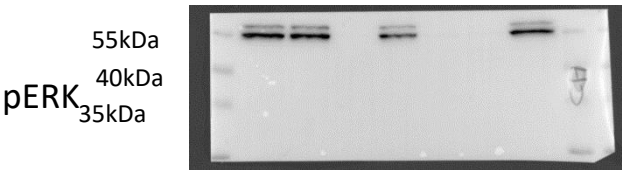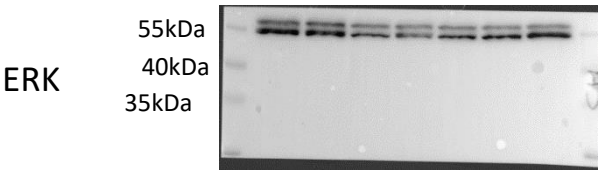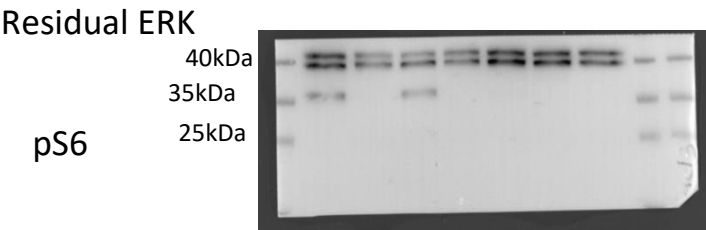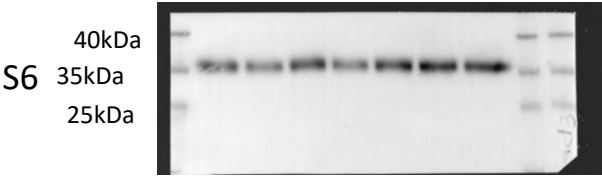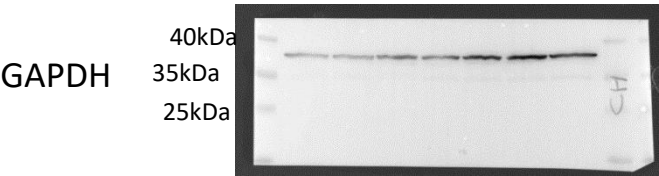

IOMM-Lee

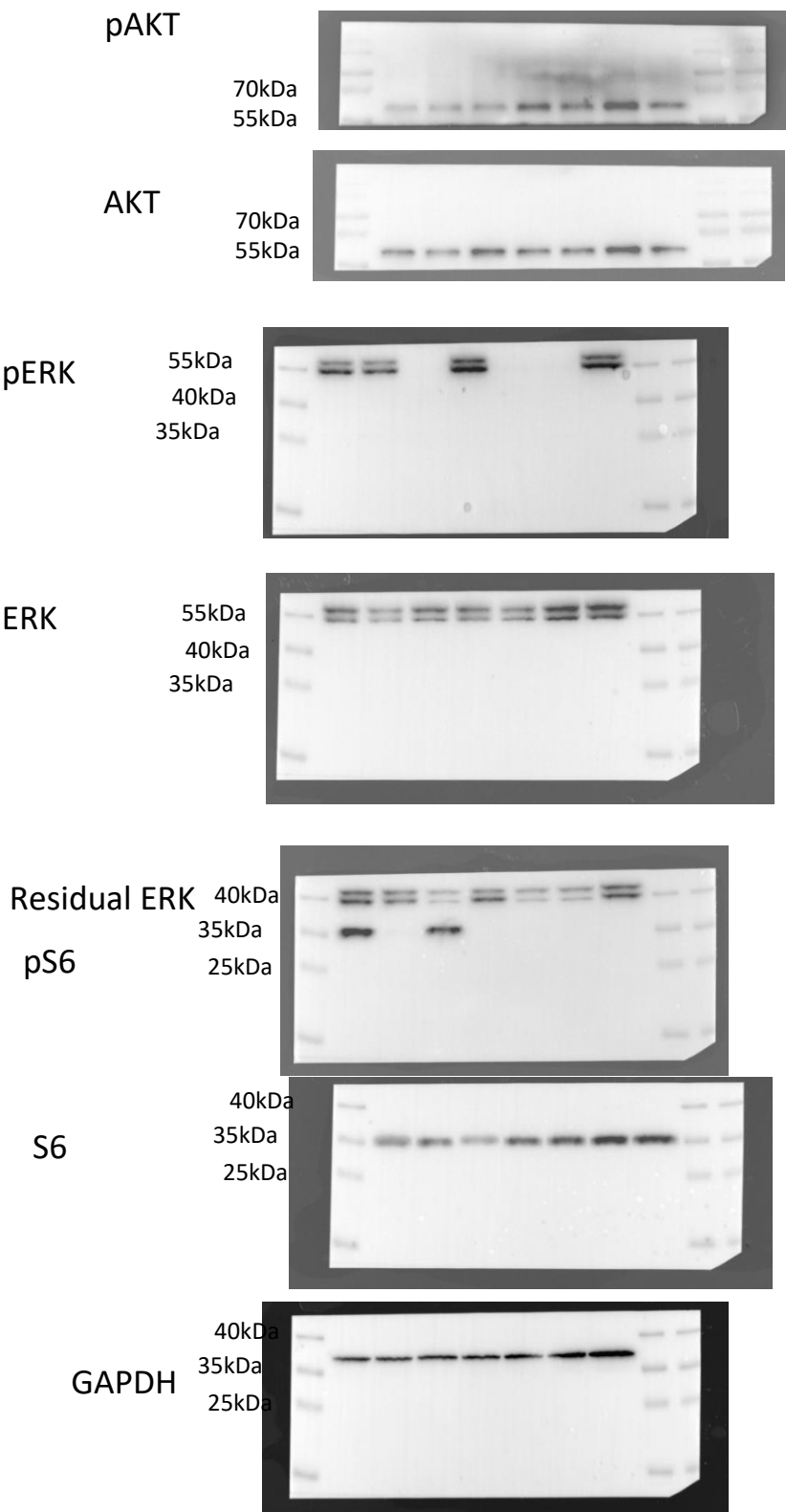

Figure 4

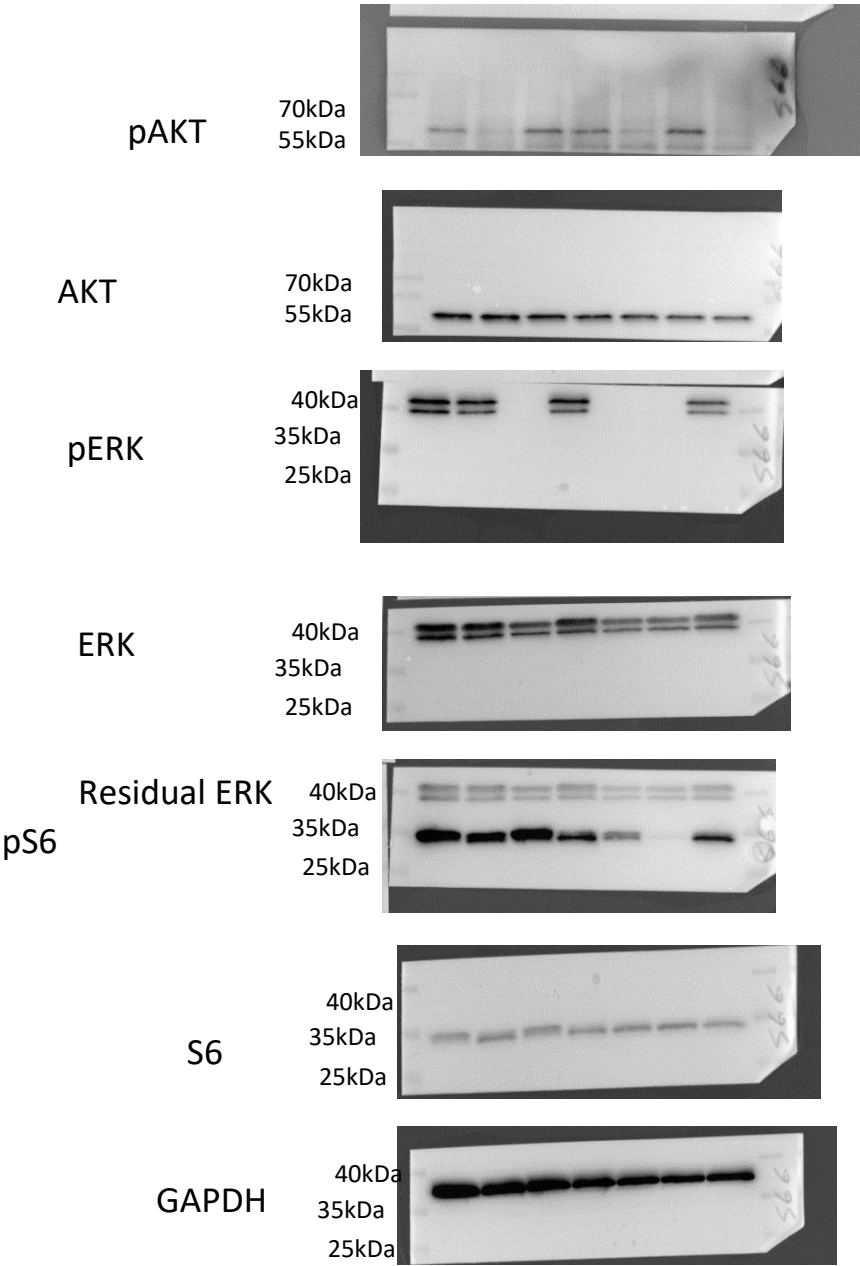

Figure 6

B

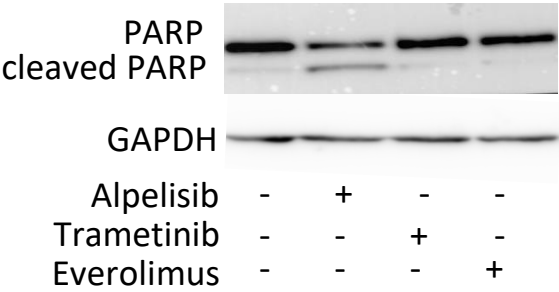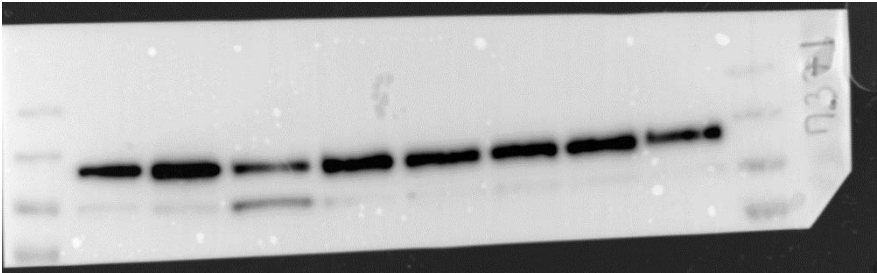

2, 3, 4 and 5 for the paper

Supplement: Supplementary file 1 [file cancers-14-04448-s001.zip › cancers-1862184-The original blots.pdf]
